# P-1508. Social Determinants of Decisions about COVID-19, Influenza, and RSV Immunization Among Predominantly Black and Latinx Communities and Households in New York City: A Cross-Sectional Analysis

**DOI:** 10.1093/ofid/ofaf695.1692

**Published:** 2026-01-11

**Authors:** Sarah E Hill, Melissa Parkinson, Amelie Foumena Nkodo, Saba Ahmed, Jenny Li, Megan McAndrew, Max Flanagan, Sarah Wiant, Henry Peralta, Jorge Benitez, Harry Reyes Nieva, Lawrence Purpura, Jason Zucker, Deborah Theodore, Magdalena E Sobieszczyk, Delivette Castor

**Affiliations:** Columbia University Irving Medical Center, Not Applicable; Columbia University, New York, New York; Columbia University Irving Medical Center, Not Applicable; Columbia University Irving Medical Center, Not Applicable; Columbia University Irving Medical Center, Not Applicable; Columbia University Irving Medical Center, Not Applicable; Columbia University Irving Medical Center, Not Applicable; Columbia University Irving Medical Center, Not Applicable; Columbia University Irving Medical Center, Not Applicable; Columbia University Irving Medical Center, Not Applicable; Columbia University Irving Medical Center, Not Applicable; Columbia University Medical Center, Brooklyn, NY; Columbia University Irving Medical Center, Not Applicable; Columbia University Irving Medical Center, Not Applicable; Division of Infectious Diseases, Department of Medicine, Vagelos College of Physicians and Surgeons, New York-Presbyterian Columbia University Irving Medical Center, New York, NY, USA, New York, New York; Columbia University Medical Center, Brooklyn, NY

## Abstract

**Background:**

In the US, COVID-19 has caused 7+ million deaths since 2020; influenza caused roughly 28,000–52,000 deaths between 2023–2024, and RSV caused about 33,000 in-hospital deaths in older adults annually. Vaccines for these viruses are highly effective in reducing illness and death. Evidence suggests that discrimination may influence vaccine uptake. In predominantly Black and Latinx communities in New York City, we measured Experiences of Discrimination (EOD), examined social determinants and measured their association with COVID-19, influenza, and RSV vaccination.

**Figure:**
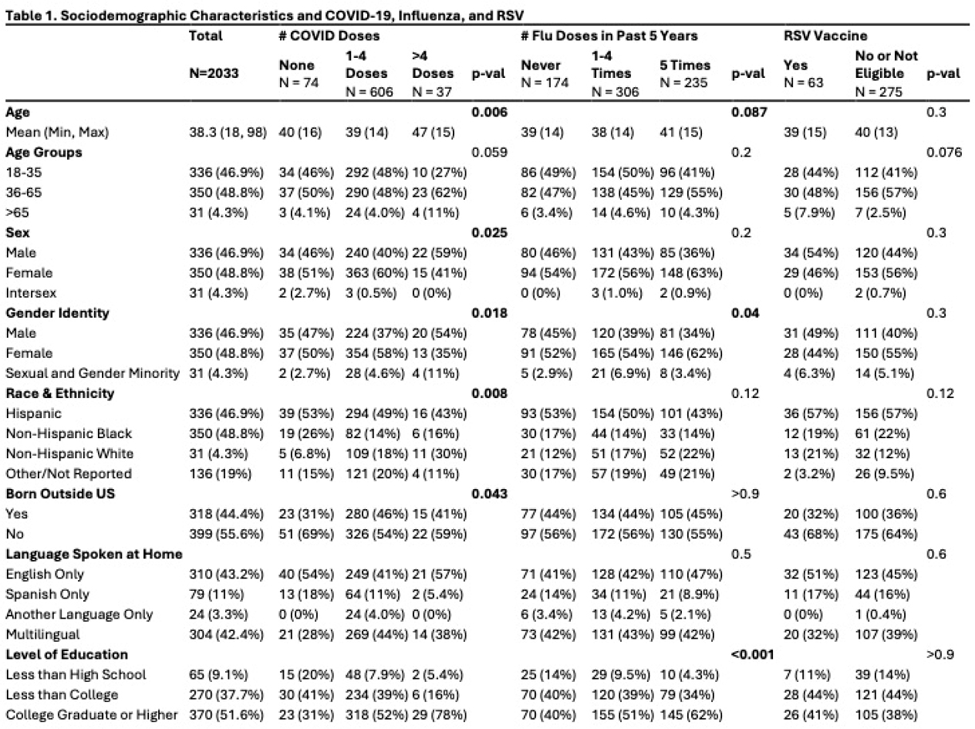

**Figure:**
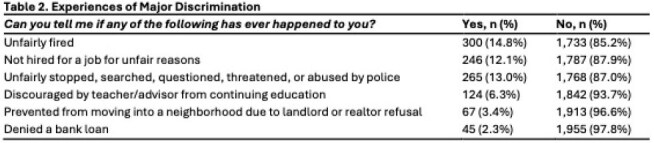
Sociodemographic characteristics and COVID-19, influenza, and RSV vaccination.

**Methods:**

This cross-sectional study recruited adults 18+ living near Columbia Medical Center. From Sep 2023 – Aug 2024, participants completed an online questionnaire collecting demographics, medical history, vaccine status, and EOD. We tabulated descriptive statistics for continuous and categorical variables and logistic regression analyses to estimate associations with vaccination.

**Figure:**
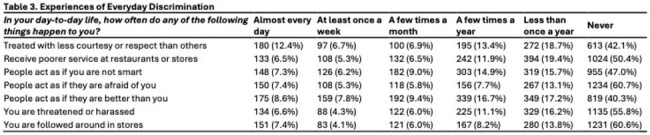

**Figure 1:**
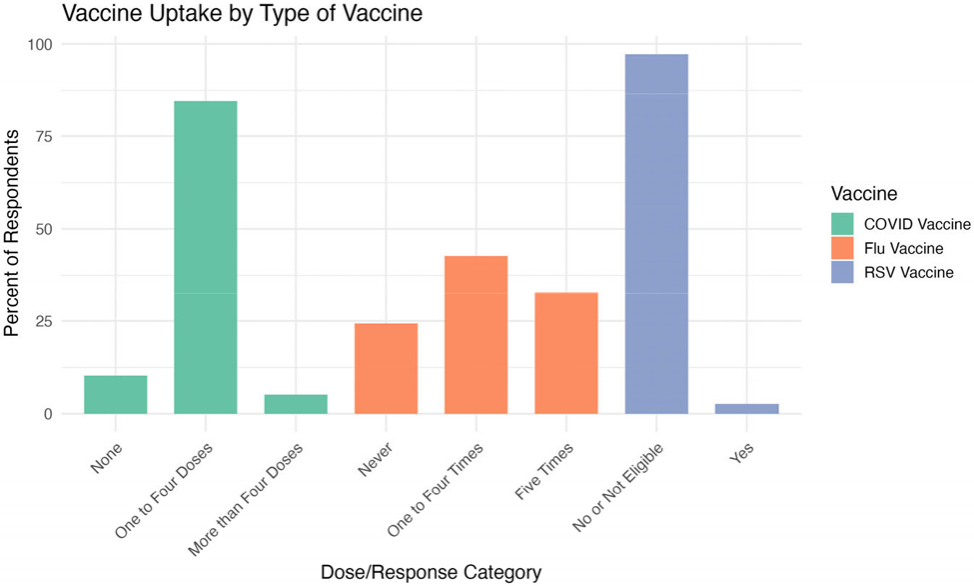
Percentage of COVID-19, influenza, and RSV vaccine doses and uptake.

**Results:**

Among 2033 participants, the mean age was 38.3 years and 58% were female. Nearly half identified as Hispanic (48.7%) and 14.9% identified as Non-Hispanic Black (Table 1). Most had received 1–4 COVID-19 vaccine doses (84.5%), 42.7% reported 1–4 influenza vaccinations in the past 5 years and 2.7% of those aged 60+ received the RSV vaccine (Figure 1). Self-reported major and everyday discrimination were low (Table 2, 3). In unadjusted tests, age, sex, gender, education, income and health insurance status were associated with vaccination (Table 1). In adjusted models, family caregivers had lower odds of receiving the COVID-19 (OR=0.14, 0.02-0.98, p=0.0363) or influenza (OR=0.18, 0.04-0.83, p=0.0276) vaccines compared to employed participants. Those with less than high school education had lower odds of influenza vaccination than those with college degrees (OR=0.32, 0.42, 1.41, p=0.004).

**Conclusion:**

Although most participants were vaccinated against COVID-19 and a many against influenza, RSV uptake was low. We also observed unexpectedly low levels of discrimination. The relatively high levels of vaccination and low levels of self-reported discrimination in this sample of predominantly minoritized, lower income individuals warrant further investigation and triangulation with other recent data from these communities.

**Disclosures:**

Lawrence Purpura, III, MD, MPH&TM, Regeneron: Grant/Research Support

